# Knowledge, Attitudes, and Practices of Moroccan Rheumatologists in the Management of Acute Septic Arthritis: Results of a National Cross-Sectional Survey

**DOI:** 10.7759/cureus.80709

**Published:** 2025-03-17

**Authors:** Nada Benzine, Hanan Rkain, Fatine Kronbi, Samya Ez-Zaoui, Chaimae Nouri, Redouane Abouqal, Jihane Belayachi, Hajjaj-Hassouni Najia, Latifa Tahiri, Fadoua Allali

**Affiliations:** 1 Rheumatology, Ayachi Hospital, Faculty of Medicine and Pharmacy, Mohammed V University, Rabat, MAR; 2 Laboratory of Biostatistics, Clinical and Epidemiological Research, Faculty of Medicine and Pharmacy, Mohammed V University, Rabat, MAR; 3 Medicine, International University of Rabat, Rabat, MAR; 4 Rheumatology, Ayachi Hospital, Ibn Sina Hospital Center, Faculty of Medicine and Pharmacy, Mohammed V University, Rabat, MAR

**Keywords:** acute septic arthritis, attitudes, knowledge, practices, rheumatologists

## Abstract

Objective

To assess the level of knowledge of Moroccan rheumatologists regarding the management of acute septic arthritis and analyze their attitudes and clinical practices toward this condition.

Methods

A descriptive cross-sectional study was conducted among Moroccan rheumatologists through a Google Forms questionnaire distributed via email. The data collected included sociodemographic characteristics of Moroccan rheumatologists, their level of knowledge, as well as their attitudes and clinical practices, assessed on a Likert scale (1 to 5).

Results

Out of the 440 questionnaires sent, 131 rheumatologists replied, corresponding to a response rate of 131 (33.58%). The average age of participants was 42.9±12.7 years, with a predominance of females (103 (84.8%)) and an average of 13.8 ± 11.3 years of experience in rheumatology. The diagnosis of septic arthritis was systematically considered in the case of acute monoarthritis, even in the absence of fever, by 122 (93.1%) rheumatologists. However, only 68 (51.9%) were aware of the association between a quick Sequential Organ Failure Assessment (qSOFA) score ≥2 and a high risk of mortality. Regarding therapeutic management, 115 (87.8%) rheumatologists preferred a third-generation cephalosporin, often combined with an aminoglycoside (110 (84%)). However, only 18 (13.7%) prescribed short-course antibiotics for small joints, and 13 (9.9%) associated this with joint lavage and/or surgical synovectomy. Furthermore, in cases of unfavorable evolution, only 19 (14.5%) routinely referred the patient to an orthopedic surgeon for surgical lavage. The main challenges identified were delayed diagnosis (90 (68.7%)) and the management of complicated cases (91 (69.5%)). To address these issues, a large majority of rheumatologists recommended the development of standardized protocols (118 (90.1%)), raising awareness among healthcare professionals about early diagnosis (116 (88.5%)), and strengthening collaboration with orthopedic surgeons (111 (84.7%)).

Conclusion

This study highlights gaps in the management of acute septic arthritis and emphasizes the need for better awareness, standardization of practices, and improved coordination with other specialties to optimize patient care.

## Introduction

Septic arthritis is defined by the presence of a cultivable microorganism in the synovium and joint cavity, causing an intra-articular inflammatory response [1]. It is a medical emergency requiring rapid intervention to prevent severe complications, including irreversible joint destruction. However, its diagnosis remains complex due to clinical manifestations that can sometimes be misleading [2,3]. Although relatively rare, this condition is associated with a mortality rate of 10% to 15% and significant morbidity [4], highlighting the importance of a multidisciplinary approach to its management.

International guidelines, particularly the 2020 French recommendations for the management of septic arthritis in native joints in adults, published by the French Society of Rheumatology (SFR) [1], provide detailed recommendations for the management of septic arthritis. However, their application to the Moroccan context faces several challenges that require specific adaptations.

Diagnostic delay is one of the main obstacles in the management of septic arthritis, with severe functional consequences. Approximately 24% to 33% of patients develop major complications, such as amputation, arthrodesis, prosthetic surgery, or severe functional deterioration [5]. This delay is often attributed to limited access to complementary investigations in certain healthcare facilities, insufficient awareness among non-specialist healthcare professionals, and a lack of knowledge about the early signs of the disease. Additionally, the absence of formalized national guidelines leads to significant variability in clinical practices, resulting in heterogeneous management depending on the facility and practitioner.

Furthermore, antibiotic therapy management poses a major challenge. Some antibiotics commonly recommended in Europe, such as rifampicin, widely advocated in the French rheumatology guidelines for the management of acute septic arthritis (SFR) [1], are only available in Morocco for the treatment of tuberculosis, limiting therapeutic options. Finally, challenges related to treatment follow-up and adherence, exacerbated by socioeconomic difficulties and disparities in healthcare access, compromise care continuity and negatively impact patient outcomes.

These challenges highlight the need to develop guidelines adapted to local realities in order to optimize the management of septic arthritis in Morocco.

The lack of specific national recommendations leads to non-homogeneous and sometimes suboptimal management of septic arthritis. Therefore, it is essential to better understand the actual practices of Moroccan rheumatologists in order to identify the main obstacles they face and propose recommendations tailored to the country’s realities.

In this context, we conducted a CAP (knowledge, attitudes, and practices) survey among Moroccan rheumatologists to assess their approach to acute septic arthritis. This study is the first of its kind at the national level to evaluate the knowledge of rheumatologists regarding this pathology. As rheumatologists, our goal was to analyze gaps in knowledge and practices, as well as the diagnostic and therapeutic challenges encountered. The data collected will not only help adapt international recommendations to local realities but also guide the development of targeted training programs. These training initiatives could be evaluated in future studies, measuring their impact before and after implementation, with the goal of optimizing the management of septic arthritis in Morocco. In the future, a study involving other medical specialties may be conducted for a possible comparison of knowledge and practices, particularly with traumatologists and infectious disease specialists.

## Materials and methods

Study type and population

We conducted a cross-sectional study of rheumatologists practicing in Morocco between April 20 and June 30, 2024.

Eligibility criteria for the study

All practicing rheumatologists in Morocco, regardless of their practice sector (public, private, or academic), were included in the study. No exclusion criteria were applied.

Sampling

This is a comprehensive study targeting all Moroccan rheumatologists. No sampling was conducted, as the questionnaire was distributed to all practicing rheumatologists via email addresses provided by the Moroccan Society of Rheumatology (SMR).

Data collection

The survey was conducted online using Google Forms, ensuring easy access and streamlined data collection. A list of 440 Moroccan rheumatologists, provided by the Société Marocaine de Rhumatologie (SMR), which maintains an up-to-date database containing their email addresses, was used to distribute the survey. An initial email explaining the purpose and process of the study, along with a link to the survey, was sent on April 20, 2024. A reminder email was sent on May 10, 2024, to encourage participation. Only responses received between April 20 and June 30, 2024, were included in the analysis.

To ensure data completeness, all questions in the questionnaire were mandatory. The questionnaire was designed to assess the knowledge, attitudes, and practices of Moroccan rheumatologists regarding the management of acute septic arthritis. It focused on key areas such as diagnostic strategies, challenges encountered in patient management, therapeutic choices (particularly with regard to antibiotic therapy), and alternatives to international protocols that may not be directly applicable in Morocco. The survey also explored barriers to effective management, including access to paraclinical examinations and appropriate treatments, and assessed the need for national recommendations. Additionally, practitioners’ expectations regarding the standardization of clinical practices were investigated.

This structured approach allowed for a comprehensive exploration of the subject matter and provided valuable insights into the management of acute septic arthritis among Moroccan rheumatologists.

Questionnaire

The survey questionnaire, detailed in Appendices, was developed based on a comprehensive review of the literature and the 2020 French recommendations for the management of septic arthritis in adult native joints (SFR) [1]. However, a specific section was added to address issues specific to the Moroccan context, considering practitioners' field experiences.

A group of five expert rheumatologists from the university hospital (doctorate and professor level) designed it. Two rounds of revisions were carried out to ensure the clarity, relevance, and alignment of the questions with the study objectives.

A pilot phase was conducted with 10 rheumatologists to assess the feasibility of the questionnaire. These pilot tests allowed refinement of the phrasing and improvement of question clarity. The average time to complete the questionnaire was 15 minutes.

The final version of the questionnaire consisted of four sections. The first section focused on demographics, including gender, age, place of practice, and professional experience of the respondents.

The second section assessed knowledge related to the diagnosis of acute septic arthritis, including its status as a medical emergency, primary site of infection, causative pathogens, mode of inoculation, qSOFA (quick Sequential Organ Failure Assessment) score, role of joint aspiration, differential diagnosis, and management of small joints.

The third section explored attitudes, focusing on rheumatologists' perceptions of the main difficulties encountered and the measures needed to improve management, particularly adaptations to the Moroccan context.

The fourth section examined practices through a clinical scenario involving suspected septic arthritis of the knee. It covered the diagnostic steps, choice of antibiotic therapy (considering local restrictions), the need for joint drainage, management of complications, and follow-up.

Evaluation scales

The knowledge of rheumatologists was assessed using a 5-point Likert scale: 1 - True, 2 - Likely, 3 - Unlikely, 4 - False, 5 - I don’t know. Attitudes and perceived difficulties were measured on a 5-point Likert scale: 1 - Strongly agree, 2 - Agree, 3 - Neutral, 4 - Disagree, 5 - Strongly disagree. Rheumatologists’ practices were explored using a 5-point Likert scale: 1 - Always, 2 - Often, 3 - Sometimes, 4 - Rarely, 5 - Never. This self-designed questionnaire was validated and adjusted by experts. Reliability analysis showed a Cronbach's α of 0.75 for the knowledge section, 0.80 for the attitude section, and 0.739 for the practice section (p<0.001), indicating good internal consistency and validity.

Statistical analysis

A descriptive analysis of the validated data was performed. Qualitative variables were presented as frequencies and percentages, while quantitative variables were expressed as means ± standard deviations (SD) or medians with interquartile ranges. All analyses were conducted using IBM SPSS Statistics for Windows, version 20.0 (IBM Corp, Armonk, NY).

Ethical considerations

The survey was approved by the Ethics Committee of Mohammed V University, Rabat (Faculty of Medicine and Pharmacy of Rabat, ethical approval reference: CERB 49-24), and was conducted in accordance with the ethical standards of the 1964 Declaration of Helsinki and its later amendments or comparable standards.

Each rheumatologist received an informational letter and a consent form explaining the study's purpose and process, along with a link to the survey. They were invited to complete a self-administered questionnaire. By responding to the questionnaire, participants consented to the use of their responses, and all data were analyzed anonymously.

## Results

Out of the 440 questionnaires sent, 131 rheumatologists completed and returned the questionnaire, corresponding to a response rate of 131 (33.5%). The average age of the participants was 42.9±12.7 years, with a majority of females (103 (78.6%)). As for their experience, 37 (28%) had been practicing rheumatology for over 20 years. In terms of their sector of activity, 44 (33.6%) worked in the private sector, while 18 (13.7%) and 30 (22.9%) worked in university hospitals and the public sector, respectively. The demographic characteristics of the participants are presented in Table 1.

**Table 1:** Sociodemographic data of participants

Variables		N=131
Age (average±SD)		42.9±12.7
Gender number (percentage,%)	Female	103 (78.6%)
	Male	28 (21.4%)
Average years of practice (average±SD)		13.8±11.4
Practice locations (percentage,%)	University Hospital Sector	18 (13.7%)
	Private sector	44 (33.6%)
	Public sector	30 (22.9%)
Experience (percentage,%)	≤5 years	46 (34.8%)
	>5, ≤10 years	19 (14.4%)
	>10, ≤20 years	30 (22.7%)
	>20 years	37 (28%)

Results of the "knowledge" section 

**Figure 1:**
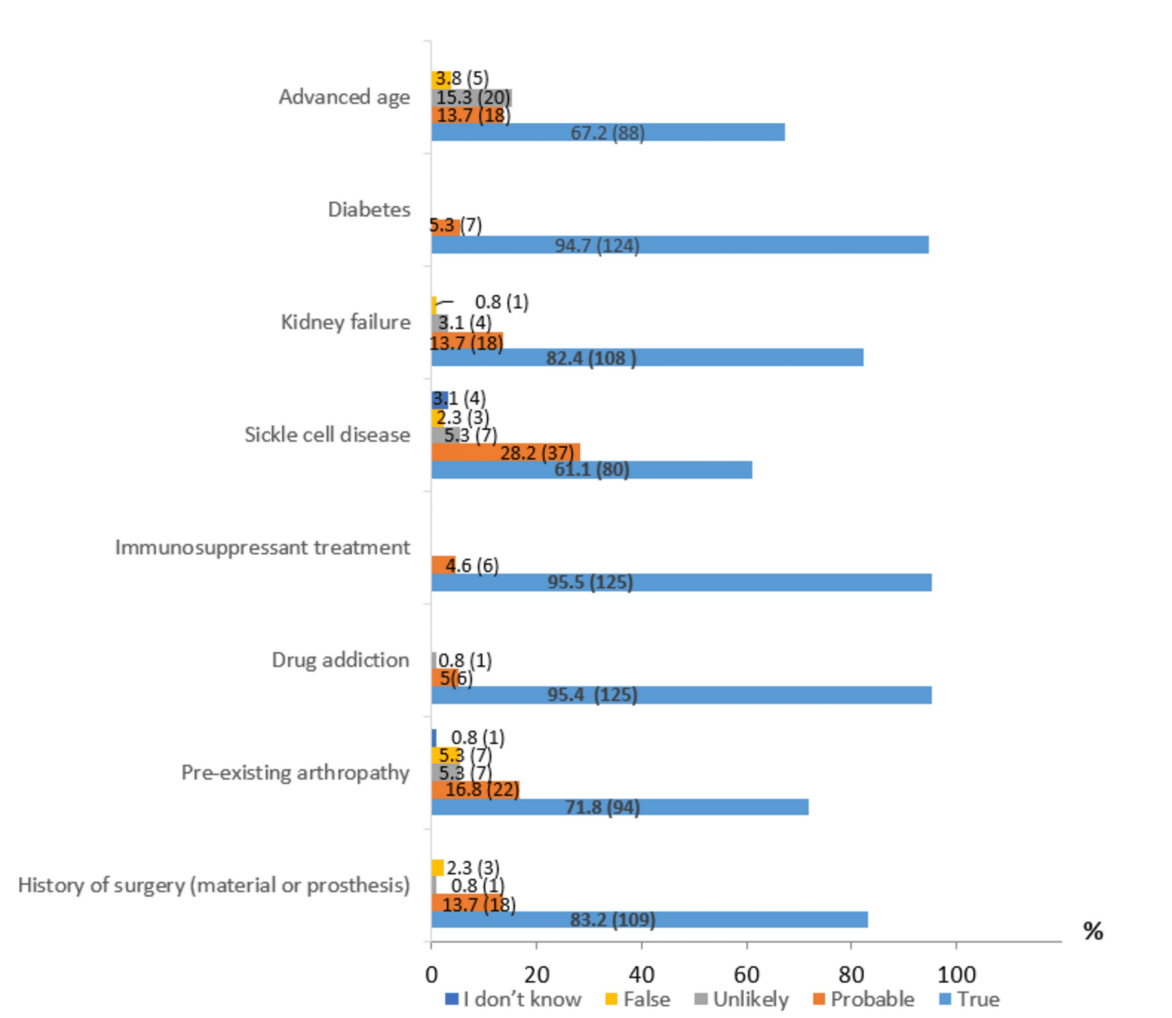
Risk factors for septic arthritis % (frequency=n): values are expressed as percentages (%), with absolute frequencies (n) indicated in parentheses.

Almost all rheumatologists (129 (98.5%)) recognize septic arthritis as a vital and functional emergency. The majority know that the knee is the most commonly affected joint (105 (80.2%)) and that Staphylococcus aureus is the most frequent infectious agent (117 (89.3%)). However, only 84 (64.1%) of rheumatologists are aware that the hematogenous route is the main mode of inoculation. In the case of acute monoarthritis, even in the absence of fever, 122 (93.1%) of respondents consider it essential to suspect septic arthritis. Regarding the qSOFA score, the criteria for respiratory rate (≥ 22 cycles/min), cognitive dysfunction, and blood pressure (≤100 mmHg) are known by 81 (61.8%), 86 (65.6%), and 80 (61.1%) of rheumatologists, respectively. However, only 68 (51.9%) know that a qSOFA score ≥2 is associated with a high risk of mortality. Joint aspiration for synovial fluid analysis is considered an essential step by 128 (97.7%) of respondents, and 98 (74.8%) acknowledge the importance of blood cultures before starting antibiotic therapy. In contrast, only 18 (13.7%) of rheumatologists are aware that septic arthritis of small joints requires a shorter course of antibiotics, and 13 (9.9%) are aware of the need for joint lavage or surgical synovectomy. The results regarding various risk factors for septic arthritis are illustrated in Figure 1.

Results from the "attitudes" section

**Figure 2:**
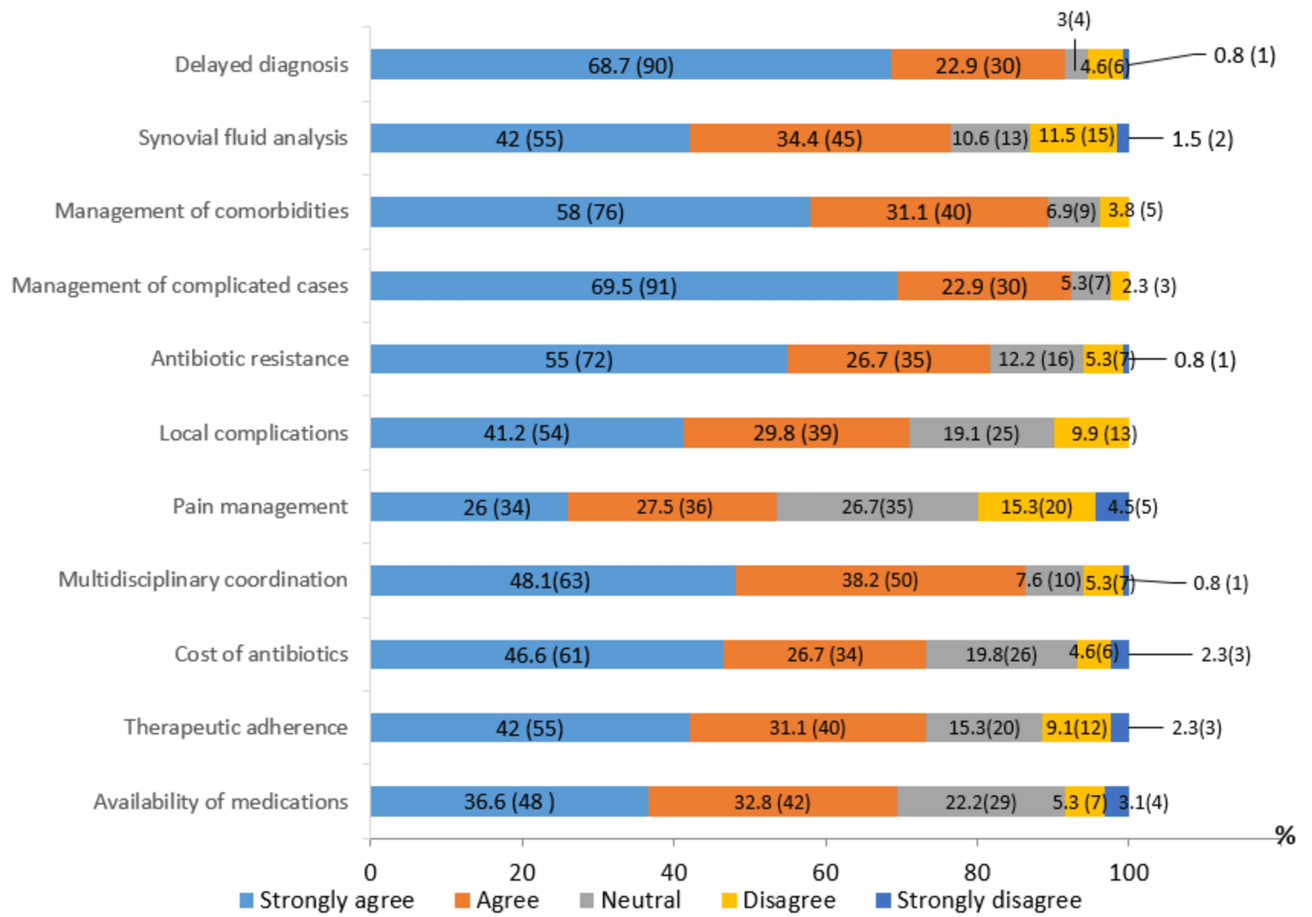
Rheumatologists' perceptions of the obstacles encountered in the management of acute septic arthritis % (frequency=n): values are expressed as percentages (%), with absolute frequencies (n) indicated in parentheses.

Among the major challenges identified, diagnostic delay (90 (68.7%)) and the management of complicated cases (91 (69.5%)) were the most reported. In terms of measures deemed necessary to improve the management of septic arthritis, Moroccan rheumatologists recommend developing standardized protocols (118 (90.1%)), improving management strategies (106 (80.9%)), raising awareness among healthcare professionals of the importance of early diagnosis (116 (88.5%)), encouraging collaboration with orthopedists (111 (84.7%)) and infectiologists (112 (85.5%)), educating patients about warning signs and the importance of follow-up (106 (80.9%)) and optimizing the care pathway (114 (87%)). However, only two-thirds of rheumatologists believe that training general practitioners can improve this care. Figure 2 illustrates rheumatologists' perceptions of the obstacles encountered in the management of acute septic arthritis.

Results from the "practices" section

**Figure 3:**
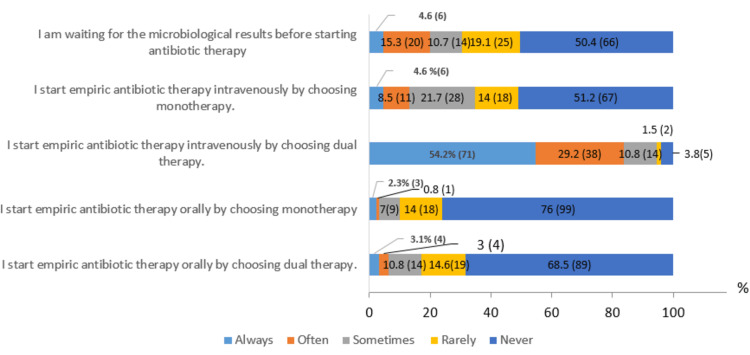
Therapeutic approach to adopt if the patient was hemodynamically stable % (frequency=n): Values are expressed as percentages (%), with absolute frequencies (n) indicated in parentheses.

In the case of acute febrile monoarthritis of the knee, 120 (91.6%) of rheumatologists always suspect septic arthritis, but only 67 (51.1%) systematically assess severity using the qSOFA score. Additionally, 79 (60.3%) immediately hospitalize the patient. Almost all rheumatologists perform joint aspiration under strict aseptic conditions (127 (97%)) and request cytological (128 (97.7%)), biochemical (90 (68.7%)), and bacteriological (129 (98.5%)) analyses. However, only 18 (13.7%) request a mycological examination. Regarding additional tests, all rheumatologists prescribe a complete blood count and CRP, while 128 (97.7%) request an erythrocyte sedimentation rate. A urine culture (105 (80.2%)), chest X-ray (104 (79.4%)), and echocardiography (67 (51.1%)) are performed less systematically. Half of the practitioners systematically request aerobic/anaerobic blood cultures, and 73 (55.7%) of rheumatologists prescribe an immobilization splint for pain relief. The survey participants were also asked about the therapeutic approach to adopt if the patient was hemodynamically stable. The results are presented in Figure 3.

For treatment, 115 (87.8%) rheumatologists prescribe a third-generation cephalosporin, often combined with an aminoglycoside (110 (84%)). Joint lavage using a needle is performed by 30 (22.9%) rheumatologists, with 47 (36.4%) performing it twice weekly. In the case of Streptococcus septic arthritis, 27 (20.6%) rheumatologists recommended a four-week course of treatment, while 49 (37.4%) opted for six weeks for Staphylococcus infections, and 40 (30.5%) recommended seven days of antibiotic therapy for *Neisseria gonorrhoeae* infections.

In case of unfavorable progression, 71 (54.2%) repeat blood cultures, and 67 (51.1%) perform another joint aspiration. Only 49 (37.4%) inoculated synovial fluid into a blood culture bottle, and 35 (26.7%) requested PCR (molecular biology) analysis of the joint fluid. Additionally, 73 (55.7%) discuss the patient's case with an infectious disease specialist. If purulent synovial fluid is found during aspiration, 60 (45.8%) of rheumatologists systematically adjust empiric antibiotic therapy in consultation with an infectious disease specialist. Furthermore, 19 (14.5%) always refer the patient to a trauma orthopedic surgeon for surgical lavage. All rheumatologists monitor patients' temperature and the majority also track pain (127 (96.9%)), joint swelling (126 (96.2%)), range of motion (104 (79.4%)), functional impact (101 (77.1%)), and muscle atrophy (85 (64.9%)).

**Figure 4:**
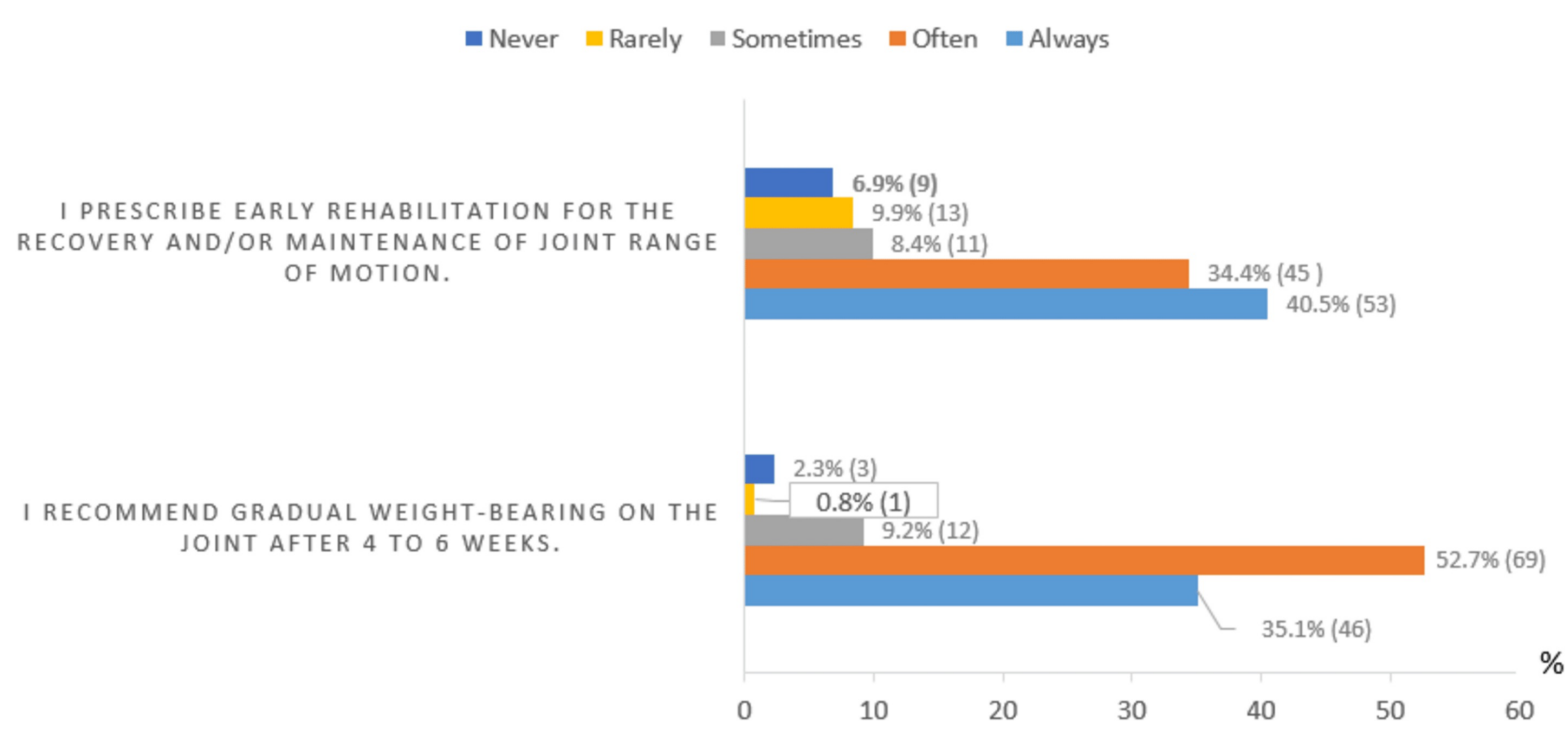
Rehabilitative management in septic arthritis % (frequency=n): Values are expressed as percentages (%), with absolute frequencies (n) indicated in parentheses.

Regarding biological follow-up, all rheumatologists request C-reactive protein (CRP) testing. Additionally, complete blood count (119 (90.8%)), erythrocyte sedimentation rate (107 (82.4%)), creatinine (84 (64.1%)), transaminases (75 (57.3%)), synovial fluid analysis (36 (27.5%)), and procalcitonin (21 (16%)) are also requested. The results of rehabilitative management in septic arthritis are presented in Figure 4.

## Discussion

This study, titled Knowledge, Attitudes, and Practices of Moroccan Rheumatologists in the Management of Acute Septic Arthritis: Results of a National Cross-Sectional Survey, represents the first national assessment of the management of this condition in Morocco. It provides an overview of Moroccan rheumatologists' knowledge, attitudes, and practices regarding acute septic arthritis, aiming to identify existing gaps and deficiencies. The findings of this survey will help to propose a standardized protocol to improve the management of this condition on a national scale.

Knowledge section 

Our results show that the majority of surveyed rheumatologists have a good understanding of acute septic arthritis, particularly its definition and the most frequently affected joint, the knee. This observation aligns with other studies, such as a systematic review by Carpenter CR, which reported that the knee is involved in 50% of acute septic arthritis cases [6]. The risk factors identified by Moroccan rheumatologists, including diabetes, advanced age, renal failure, sickle cell disease, pre-existing arthropathy, and immunosuppressive treatments, are also consistent with findings from similar studies. For instance, a study conducted in Amsterdam on more than 7000 patients found that risk factors included age over 80, diabetes, rheumatoid arthritis, and recent joint surgery. An increased prevalence of septic arthritis in hemodialysis patients was also observed. Regarding post-arthroscopic septic arthritis, its prevalence was estimated at approximately 14 per 10000 procedures (0.14%) [7,8].

Staphylococcus aureus is identified as the primary causative pathogen by Moroccan rheumatologists, a finding supported by other studies. A retrospective study conducted by Stephen McBride showed that Staphylococcus aureus was the most frequently isolated organism, with 13% of cases involving methicillin-resistant strains (MRSA), followed by streptococci and Gram-negative bacilli [9]. However, only 78 (60%) of rheumatologists were familiar with the parameters of the qSOFA score and its association with mortality risk, highlighting the need to improve knowledge of this score.

It is important to clarify that the qSOFA (quick Sequential Organ Failure Assessment) is a simple clinical score used to identify patients at risk of severe sepsis or septic shock. It is based on three criteria: a respiratory rate ≥22/min, a systolic blood pressure ≤100 mmHg, and an altered mental status (Glasgow Coma Scale <15). A qSOFA score above 2 indicates a high risk of complications, requiring urgent management. In acute septic arthritis, qSOFA is a useful tool for detecting systemic involvement and guiding rapid decision-making [10].

Another point of divergence concerns the therapeutic management of septic arthritis in small joints. Only 18 (13.7%) of rheumatologists knew that a short course of antibiotics was appropriate, and fewer than 13 (10%) were aware of the importance of combining surgical lavage or synovectomy with antibiotic therapy, contrary to current literature recommendations [11]. An open-label randomized study demonstrated the non-inferiority of a two-week antibiotic regimen compared to a four-week regimen in this specific context [12].

Attitudes section

The management of acute septic arthritis in Morocco faces several challenges, including diagnostic delays, the treatment of complicated cases, and the management of comorbidities. These obstacles are well-documented in the literature, as septic arthritis is a severe infection that threatens both vital and functional prognosis. Some studies report that one-year mortality can reach 10%, while local septic complications and functional sequelae may affect up to 30% of cases [13-15].

Approximately half of Moroccan rheumatologists consider antibiotic resistance as a major issue in the management of septic arthritis, a concern also highlighted in several international studies. For instance, in a study by Lee et al., methicillin-resistant Staphylococcus aureus (MRSA) was identified as the causative agent in 39% of Staphylococcus aureus-related arthritis cases [16].

Concerning the measures to improve the management of septic arthritis, 118 (90.1%) rheumatologists recommended the development of standardized management protocols. This suggestion addresses a crucial need, particularly given the lack of specific local guidelines. Even at the international level, existing recommendations are often expert-based consensus with a low level of evidence. Additionally, 116 (88.5%) rheumatologists supported raising awareness among healthcare professionals about the importance of early diagnosis of septic arthritis. This highlights the need for rapid intervention, as joint destruction can begin as early as 24 to 48 hours after infection onset [13].

Practices section

The initial management of acute septic arthritis by Moroccan rheumatologists generally involves hospitalization in 79 (60%) cases. However, only half of the rheumatologists assessed severity signs using the qSOFA score, despite the 2020 recommendations of the FSR on the management of septic arthritis in native joints in adults, which advocates for its use as soon as septic arthritis is suspected [1].

The majority of rheumatologists conducted a thorough interview and a complete clinical examination, followed by a joint aspiration performed under strict aseptic conditions for cytological and bacteriological analysis. This approach aligns with practices reported in other studies, such as a cohort study conducted by Paul Stirling et al. [17].

For the paraclinical evaluation, nearly half of the rheumatologists requested aerobic-anaerobic blood cultures as soon as septic arthritis was suspected, in line with global recommendations, particularly those of Habib G et al. and Baddour LM et al., who advocate for performing at least two sets of aerobic-anaerobic blood cultures before initiating any antibiotic therapy [17-19].

Regarding therapeutic management, 89 (68%) rheumatologists immediately started probabilistic antibiotic therapy, even in the absence of signs of severity. This practice contradicts the recommendations of the SFR (Société française de rhumatologie) on the management of septic arthritis in native joints in adults, which recommend waiting for microbiological results before initiating antibiotic treatment [1]. While the majority of Moroccan rheumatologists choose a third-generation cephalosporin as the initial antibiotic therapy, the literature suggests that treatment should be adjusted based on the patient's age.

For example, a study conducted by Guillaume Coiffier recommends the use of a first-generation injectable cephalosporin (C1G) for patients under 70 years old. However, for patients over 70 years old, it is recommended to use a third-generation injectable cephalosporin (C3G) due to a higher frequency of gram-negative bacilli (BGN) in this population [20].

In cases of septic arthritis caused by Streptococcus, only 27 (20.6%) rheumatologists recommended a four-week treatment duration, while 49 (37.4%) opted for six weeks for Staphylococcus infections. Additionally, only 40 (30.5%) recommended a seven-day antibiotic course for Neisseria gonorrhoeae infections. However, according to the recommendations of the FSR on the management of septic arthritis in native joints in adults, antibiotic therapy should typically last between four and six weeks. This duration depends on factors such as the timing of diagnosis, the initiation of antibiotic therapy, the patient's clinical response, and the type of bacteria involved [1]. A four-week treatment for Streptococcus septic arthritis and a six-week duration for Staphylococcus infections have been suggested [1].

Only 30 (23%) Moroccan rheumatologists perform needle joint lavage in the management of acute septic arthritis, despite several studies recommending this practice. The goal of joint lavage is to reduce the bacterial load within the joint, which enhances the effectiveness of antibiotics and helps limit cartilage degradation [21].

In cases of unfavorable evolution, only 19 (14.5%) Moroccan rheumatologists refer the patient to an orthopedic-traumatologist for an arthroscopic lavage, which does not align with various studies that recommend surgical lavage when purulent fluid persists and/or positive cultures remain after five to seven days of appropriate antibiotic therapy [22].

52 (40%) rheumatologists prescribed rehabilitation management, in line with the recommendations of the FSR on the management of septic arthritis in native joints in adults. These recommendations advocate for early rehabilitation to maintain and improve the passive joint range of motion while considering the patient's pain level [1].

It is, therefore, imperative that rheumatologists integrate rehabilitation care more extensively into their clinical practice to optimize the functional outcomes for patients with joint problems and to promote their optimal recovery.

In regard to clinical-biological follow-up, most rheumatologists monitor the patient's temperature, pain, joint swelling, joint range of motion, and functional impact, which aligns with the recommendations of the FSR on the management of septic arthritis in native joints in adults [1].

For biological monitoring, most rheumatologists ensure follow-up that includes CRP, hemogram, and sedimentation rate to assess the infectious syndrome, as well as liver transaminases and renal function tests to monitor the tolerance of antibiotic therapy. This approach is consistent with the recommendations of the SFR on the management of septic arthritis in native joints in adults [1].

It is, therefore, essential that practitioners maintain this rigorous clinical-biological follow-up approach to ensure optimal management of septic arthritis and guarantee satisfactory clinical outcomes for patients.

Our study has several notable strengths. It is the first comprehensive national survey conducted among Moroccan rheumatologists, offering deep insights into the management of acute septic arthritis across the country. Through the careful evaluation of knowledge, attitudes, and practices, our study provides a holistic understanding of the current landscape surrounding acute septic arthritis among rheumatologists.

Furthermore, our findings address critical issues such as the evaluation parameters using the qSOFA score, diagnostic methods, and therapeutic and rehabilitative management of acute septic arthritis. These perspectives provide invaluable guidance for clinical practice, potentially enhancing the quality of care provided to patients suffering from acute septic arthritis.

We believe that our study makes a significant contribution to the existing literature on acute septic arthritis, particularly in regions like Morocco, which have been underrepresented in research. It helps fill gaps in knowledge and raises awareness. Our study is a vital step toward a better understanding and management of acute septic arthritis, laying the groundwork for future research and interventions aimed at improving patient care and outcomes.

However, our study has some limitations. Despite our efforts to obtain a representative sample, the participation of 131 rheumatologists may not fully reflect the diversity of practices and perspectives across the country. We chose to start with rheumatologists because they play a key role in the management of joint diseases and are often involved in the diagnosis and follow-up of patients with acute septic arthritis. However, the fact that this survey was conducted exclusively among this specialty represents a limitation. We were unable to include other specialists involved in the management of acute septic arthritis, such as orthopedic surgeons and infectious disease specialists, which restricts the scope of our conclusions. Moreover, the reliance on self-reported data introduces a risk of bias or inaccuracies, particularly regarding practices and attitudes. Finally, the cross-sectional nature of our study limits our ability to establish causal relationships or track changes in knowledge, attitudes, and practices over time.

## Conclusions

In conclusion, our survey highlights both the strengths and the gaps in the management of acute septic arthritis by Moroccan rheumatologists. Our results demonstrate an urgent need to develop standardized management protocols adapted to the Moroccan context, as well as to improve the awareness and training of rheumatologists. Furthermore, increased collaboration among rheumatologists, infectious disease specialists, and orthopedic-traumatologists appears essential to optimize the management of this condition. This study represents an important step toward a better understanding and management of acute septic arthritis, with crucial implications for future research and interventions aimed at improving the quality of care and patient outcomes.

## Appendices

Questionnaire

Physician's Identity

Age (in years):

Sex:

o   Male

o   Female

Years of experience in rheumatology:

You are a:

o   Rheumatologist in the public sector

o   Rheumatologist in the private sector

o   Rheumatologist in the CHU sector

A) Knowledge

Septic arthritis is a life- and function-threatening emergency:

o   True

o   Probable

o   Unlikely

o   False

o   Don't know  

Septic arthritis more often affects the knee:

o   True

o   Probable

o   Unlikely

o   False

o   Don't know

Among the following proposals, please tick the corresponding box (Table 2): 

**Table 2:** Among the following proposals, please tick the corresponding box

Risk factors for septic arthritis	True	Probable	Unlikely	False	Don't know
Advanced age					
Diabetes					
Renal failure					
Sickle cell disease					
Immunosuppressive therapy					
Drug abuse					
Pre-existing joint disease					
History of surgery					

Among the infectious agents involved in septic arthritis, Staphylococcus aureus is the most common:

o   True

o   Probable

o   Unlikely

o   False

o   Don't know  

The main mode of inoculation for septic arthritis is the hematogenous route:

o   True

o   Probable

o   Unlikely

o   False

o   Don't know

Septic arthritis should be considered in the presence of any acute non-febrile mono-arthritis:

o   True

o   Probable

o   Unlikely

o   False

o   Don't know

A respiratory rate ≥22 is considered in the evaluation of the quick Sequential Organ Failure Assessment (qSOFA) score:

o   True

o   Probable

o   Unlikely

o   False

o   I don’t know

Cognitive dysfunction (confusion, disorientation, Glasgow Coma Scale (GCS) <15) is considered in the qSOFA score evaluation:

o   True

o   Probable

o   Unlikely

o   False

o   I don’t know

A blood pressure ≤100 mmHg is considered in the qSOFA score evaluation:

o   True

o   Probable

o   Unlikely

o   False

o   I don’t know

A qSOFA score ≥2 indicates a sepsis-related mortality risk of ≥10%:

o   True

o   Probable

o   Unlikely

o   False

o   I don’t know

Joint aspiration for cytobacteriological analysis of synovial fluid is essential before initiating antibiotic therapy:

o   True

o   Probable

o   Unlikely

o   False

o   I don’t know

Blood cultures must be performed before initiating antibiotic therapy:

o   True

o   Probable

o   Unlikely

o   False

o   I don’t know

Septic arthritis of small joints (fingers and wrist) requires a shorter antibiotic course (2 weeks):

o   True

o   Probable

o   Unlikely

o   False

o   I don’t know

Septic arthritis of small joints (fingers and wrist) often requires, in addition to antibiotic therapy, surgical lavage/synovectomy:

o   True

o   Probable

o   Unlikely

o   False

o   I don’t know

B) Attitudes

What are the complex challenges in the management of septic arthritis? (Table 3) 

**Table 3:** What are the complex challenges in the management of septic arthritis?

	Strongly agree	Agree	Disagree	Strongly disagree	Neutral
Delayed diagnosis					
Synovial fluid analysis					
Management of comorbidities					
Handling complicated cases					
Antibiotic resistance					
Local complications					
Pain management					
Multidisciplinary coordination					
Cost of antibiotics					
Therapeutic adherence					
Availability of medications					

In your opinion, what measures do you consider necessary to improve the management of septic arthritis? (Table 4)

**Table 4:** What measures do you consider necessary to improve the management of septic arthritis?

	Strongly agree	Agree	Disagree	Strongly disagree	Neutral
Develop standardized management protocols					
Train general practitioners					
Raise awareness among healthcare professionals about the importance of recognizing septic arthritis signs early for timely diagnosis					
Strengthen collaboration between rheumatologists and orthopedic surgeons					
Strengthen collaboration between rheumatologists and infectious disease specialists					
Educate patients about warning signs, the importance of treatment, and medical follow-up					
Improve the patient care pathway					

C) Practices

Based on your experience, please answer the following questions using the following clinical case example:

Mr. M, a 54-year-old man with no significant medical history, presents with acute right knee pain associated with a fever of 39°C, evolving over three days.

I suspect septic arthritis:

o   Always

o   Often

o   Sometimes

o   Rarely

o   Never

I assess severity using the qSOFA score:

o   Always

o   Often

o   Sometimes

o   Rarely

o   Never

I hospitalize the patient:

o   Always

o   Often

o   Sometimes

o   Rarely

o   Never

I do a thorough interrogation:

o   Always

o   Often

o   Sometimes

o   Rarely

o   Never

I do a complete clinical examination:

o   Always

o   Often

o   Sometimes

o   Rarely

o   Never

I perform joint aspiration under strict aseptic conditions :

o   Always

o   Often

o   Sometimes

o   Rarely

o   Never

I systematically request a biochemical analysis of synovial fluid:

o   Always

o   Often

o   Sometimes

o   Rarely

o   Never

I systematically request a cytological analysis of synovial fluid:

o   Always

o   Often

o   Sometimes

o   Rarely

o   Never

I systematically request a bacteriological analysis of synovial fluid:

o   Always

o   Often

o   Sometimes

o   Rarely

o   Never

I systematically request a mycological analysis of synovial fluid:

o   Always

o   Often

o   Sometimes

o   Rarely

o   Never

I systematically request two sets of aerobic/anaerobic blood cultures:

o   Always

o   Often

o   Sometimes

o   Rarely

o   Never

Among the following additional tests, which do you request regularly? (Table 5)

**Table 5:** Among the following additional tests, which do you request regularly?

	Always	Often	Sometimes	Rarely	Never
Complete blood count (CBC)					
Erythrocyte sedimentation rate (ESR)					
C-reactive protein (CRP)					
Urinalysis (ECBU)					
Chest X-ray					
Echocardiography					

I prescribe early passive immobilization for pain relief:

o   Always

o   Often

o   Sometimes

o   Rarely

o   Never

Following Mr. M.’s clinical examination, he was found to be hemodynamically stable, with a systolic blood pressure of 110 mmHg, a respiratory rate of 25/min, and a Glasgow Coma Score of 15/15. A synovial fluid aspiration was performed, and initial laboratory tests were requested.

I wait for microbiological results before starting antibiotic therapy:

o   Always

o   Often

o   Sometimes

o   Rarely

o   Never

I start empirical intravenous antibiotic therapy with monotherapy:

o   Always

o   Often

o   Sometimes

o   Rarely

o   Never

I start empirical intravenous antibiotic therapy with dual therapy:

o   Always

o   Often

o   Sometimes

o   Rarely

o   Never

I start empirical oral antibiotic therapy with monotherapy:

o   Always

o   Often

o   Sometimes

o   Rarely

o   Never

I start empirical oral antibiotic therapy with dual therapy:

o   Always

o   Often

o   Sometimes

o   Rarely

o   Never

Which antibiotic therapy do you choose?

I consider a treatment duration of 4 weeks in case of septic arthritis due to Streptococcus:

o   Always

o   Often

o   Sometimes

o   Rarely

o   Never

I consider a treatment duration of 6 weeks in the case of Staphylococcus:

o   Always

o   Often

o   Sometimes

o   Rarely

o   Never

I consider a total treatment duration of 7 days in the case of Neisseria gonorrhoeae arthritis:

o   Always

o   Often

o   Sometimes

o   Rarely

o   Never

I perform needle joint lavage:

o   Always

o   Often

o   Sometimes

o   Rarely

o   Never

I prescribe early rehabilitation to restore and/or maintain joint range of motion:

o   Always

o   Often

o   Sometimes

o   Rarely

o   Never

I ensure clinical follow-up of the patient by considering the following elements (Table 6): 

**Table 6:** I ensure clinical follow-up of the patient by considering the following elements

	Always	Often	Sometimes	Rarely	Never
Temperature					
Pain					
Joint range of motion					
Joint swelling					
Muscle atrophy					
Functional impact					

I ensure the patient's biological follow-up by considering the following elements (Table 7):

**Table 7:** I ensure the patient's biological follow-up by considering the following elements

	Always	Often	Sometimes	Rarely	Never
Complete blood count (CBC)					
Erythrocyte sedimentation rate (ESR)					
C-reactive protein (CRP)					
Renal function					
Transaminases					
Synovial fluid analysis					
Procalcitonin					

Assuming an unfavorable evolution, on the third day of empirical antibiotic therapy, Mr. M remains febrile. The results of the synovial fluid culture are negative, as are the blood cultures, with an elevated C-reactive protein.

What would be your next steps in this case? (Table 8)

**Table 8:** What would be your next steps in this case?

	Always	Often	Sometimes	Rarely	Never
I repeat the blood cultures					
I repeat the joint aspiration					
I inoculate the synovial fluid onto a blood culture bottle					
I request a PCR (molecular biology) test on the synovial fluid immediately					

The second joint aspiration reveals purulent fluid, what would be your next steps:

I change the initial antibiotic therapy in consultation with the infectious disease specialist:

o   Always

o   Often

o   Sometimes

o   Rarely

o   Never

I refer the patient to an orthopedic surgeon for surgical lavage:

o   Always

o   Often

o   Sometimes

o   Rarely

o   Never
